# Ways and means to comfort people at the end of life: how is the nurse a privileged player in this process?

**DOI:** 10.1177/26323524231182730

**Published:** 2023-07-12

**Authors:** Raquel Alexandra Machado Pereira, Patrícia Cruz Pontífice Sousa Valente Ribeiro

**Affiliations:** Machado Pereira Universidade Católica de Lisboa, Palma de Cima, Edificio Reitoria, 1649-023 Lisbon, Portugal; Universidade Católica de Lisboa, Lisbon, Portugal

**Keywords:** comfort, ethnography, nursing, palliative care

## Abstract

**Background:**

Comfort is a necessity throughout life, and it is a key element in the practice of nursing care for the patient at the end of life. A particular human need and a state related to the experience and culture of the person at the end of life constitute the target of attention and nursing intervention, being a very relevant indicator of the quality of health care. This article is part of a doctoral study in the field of comfort in a palliative care unit, and these are some of the partial results that emerged.

**Objectives:**

To understand the ways and means of comfort perceived by the person at the end-of-life hospitalized in a palliative care unit, their family, and health staff as well as the value of the nurse in this process.

**Design:**

Qualitative study using an ethnographic approach.

**Methods:**

We conducted semistructured interviews with 18 patients at the end of life and their matched significant family members (18) and 21 health professionals. We also conducted participant observation of care situations.

**Results/Discussion:**

The ways and means of providing comfort are centered on strategies developed by the entire multidisciplinary team. During this whole process, one of the categories that emerged from the ethnography was the nurse as a privileged player, representing an absolutely essential role in all phases. The results revealed that nurses play a very important role in end-of-life comfort, which is based on a predisposition for end-of-life care (active listening, empathy, congruence, and biographical narrative) and focused attention (global care, attention to detail, family support, and opposition to therapeutic obstinacy).

**Conclusions:**

The different ways and means of providing comfort aim to increase care, relieve, and invest in potential different forms of comfort and nurses are recognized by all those involved in this process as someone essential to providing comfort care.

## Introduction

Comfort is an important concept and of enormous value for the nursing profession,^1,2^ constituting a key element in the provision of care to all people but particularly to the person at the end of life.

Comfort is promoted, among other things, through nursing interventions, but also through other professionals and the family itself. Comforting is a care factor and a nurse’s competence, whereby, understanding the ways and means of comforting the person at the end of life hospitalized in a palliative care unit is of significant importance.

It is also important to understand the meaning of comfort, as well as the ways and means of providing comfort, to define effective interventions.

We know that only through research we can develop knowledge that leads to the effective inclusion of comfort in the health care of these specific units.^1,3^ These investigations are intended to identify an existing knowledge base and the need to develop and mobilize additional knowledge and skills to improve the provision of care to people at the end of life in a hospital context. By analyzing the literature, it appears that comfort and the relief of discomfort can have different meanings for different individuals and the lack of an effective understanding of the aspects that are inherent in certain situations justifies its exploration. From all the definitions reviewed, the search for the satisfaction of the needs of the person as a unique being is common to all. Also common to all of them is the need to relieve physical, emotional, spiritual, or social discomfort of the person at the end of life. The aforementioned studies reveal that hospitalization, even if in a palliative care unit, can disturb all these dimensions affecting the way people experience the end-of-life process.

This article intends to understand the ways and means of comfort perceived by the person at the end-of-life hospitalized in a palliative care unit, their family and health staff, as well as the value of the nurse in this process. Also, this article is part of a doctoral study in the field of comfort in a palliative care unit and these are some of the partial results that emerged.

The construction of comfort as a process is based on the defining characteristics of the actors in context influencing their ways and means of actions, contextualized in the individuality and uniqueness of each one of them and related to the patterns of thinking, feeling, and acting.^4,5^ The ways and means of comforting action assume the most varied dimensions and we consider it essential to describe and analyze them. We know that in any disease progression that implies hospitalization, the person’s fragility is accentuated, which translates into a greater susceptibility to suffering and an increase in discomfort. In the case of palliative care units, these discomforts can be very intense since we are facing a situation of an incurable disease that frequently generates a large amount of suffering and discomfort. Comforting is a complex act that involves much more than only relieving pain or ensuring proper nutrition. It also includes attention to all the dimensions of the human being.^5,6^ Care, to be comforting, has to be tailored to the person, responding to their uniqueness and needs, which may be a challenge for all professionals in this area.

The observation of everyday interactions put us in contact with a reality that, although we think we know it through our professional activity, it turned out to be surprising during the investigation process. Due to the diversity of actors present in everyday life and the current organizational model, we perceive that the actors present in the context conditioned and were conditioned through the encounter of underlying cultures.

## Methods

### Adopted definitions and framework

This is a qualitative study using an ethnographic approach. Among the qualitative research approaches used in nursing and in the field of health to obtain data related to the culture of a group and the way in which health professionals exercise their autonomy in their daily work, ethnography and ethno-nursing stand out.^7–9^ These methods are powerful ways of obtaining ‘facts, feelings, worldviews and other types of data that reveal the real world, truths and people’s ways of life’, allowing the understanding of beliefs and values.^7^ We followed the model proposed by James Spradley^8,9^ and we have chosen this method because it allows us to make sense of the lived experience, the way in which the situation is culturally experienced, interpreting it in its natural context from the different points of view of the participants to obtain meaningful contents that allow describing and understanding human experiences.

### Methodological procedures

Research based on ethnography allows one to look beyond the evidence, looking for the underlying and implicit meanings that people, as members of a subculture, attach to their practice. What interests the ethnographer are the common experiences lived in a group endowed with a culture in which the human being (person at the end of life, family members, and caregivers) is seen as an open being who interacts with himself, others, and the environment.^10^

As foreseen in ethnography, we used several data collection methods that complement each other. We started with participant observation, as it allows us to understand the consonance between what people say they do and what they actually do because, sometimes, people are not aware of the subtleties of what happens in the interactions between them. We also carried out semistructured interviews with all the participants: people at the end of life, family, and health professionals, based on a script that was submitted to a pretest with one patient, one health professional, and one family member who were not part of the participants in our study. With this, we aimed to test the clarity and understanding of the questions included in the interview.

### Study setting

Based on the purpose of the research, a palliative care unit of a hospital on the outskirts of Lisbon was chosen as the setting for the study. The choice of location was intentional and based on practical and methodological reasons: being geographically accessible; being a palliative care unit integrated within a hospital center, with people at the end of life, the vast majority in need of symptom control, whether physical or emotional; and finally, because this particular unit is one of the few in Portugal that integrates a multidisciplinary team made up of doctors (including a physiatrist), nurses, operational assistants, superior social service technician, therapists, psychologist, dietician, and spiritual assistant.

To define the participants, for the sample selection, we defined the following inclusion criteria: being hospitalized in the acute care unit with an incurable, chronic disease that causes suffering; being aware and oriented to be able to respond orally to the applied questions; freely consent to participate in the study. For each patient interviewed, their significant family member was also interviewed. The selection of people at the end of life and family members were carried out by the main researcher, based on prior knowledge of hospitalized people and information obtained from nurses and other professionals, or by consulting data in clinical files. We had to make sure that the patient and family members both wanted to participate in the study before the interviews, if one of them did not accept to participate, they would both be excluded from the sample. Finally, all healthcare professionals in the unit were interviewed after consent.

With regard to observation, we sought access to the field and paid attention to the experiences lived by the actors in the events and situations that were taking place over time.

### Participants

The actors involved in our study include a total of 57 participants among people at the end of life, professionals, and family members: 18 patients at the end of life; 18 matched family members, and 21 health professionals. It was absolutely necessary that the patient and family members accept to participate since we intended to ask questions related to the situation to both of them and then compare the answers.

The selection was carried out by the researchers, with the collaboration of the nurses present in the respective shifts, taking care to verify the availability of each patient through an initial presentation dialogue. Regarding the state of consciousness and the ability to respond orally, in the presence of doubts, we resorted to the help of nurses and also, we used the Glasgow Coma Scale to assess orientation and ability to answer questions effectively.

The final composition of the sample was determined by data saturation, as well as the achievement of information redundancy, in which new information came to confirm the previous ones, not objectively adding new data.^11^

### Data collection methods

Data collection was carried out between October 2019 and January 2021, for a total of about 16 months in interactions, to fulfill the participant observation phases and interview all informants. A total of about 137 h were spent in the field, resulting in 78 field notes and 57 recorded interviews (all participants were observed and interviewed: patients, family members, and health professionals). During this period, we stayed in the field 1–5 h per day, in scattered periods, distributed in the three shifts and at different times of the day, as we considered important to observe the participants at different times. However, we favored the morning and afternoon shifts as they have a larger number of participants on the ground at the same time and it is easier to observe their interactions.

With regard to participant observation, before moving into the field, we prepared a flexible observation guide for our observation, open and wide enough not to focus only on what it anticipates, but also on everything that may arise around it and that is of interest for the study, even if it is not included in the guide. This way, very general aspects to be observed were foreseen, which refer to what Spradley^8^ calls ‘descriptive observation’. It was intended to involve the researcher in the context of the palliative care unit to get to know all the actors in it, observing the participants and allowing them to become familiar with the researcher (we observed all the activities of the staff, the patients and families: conversations, family conferences, meals, interactions, shift changes, among others). In a second moment, we carried out an observation with some participation, we encouraged interaction with the participants by recording their reactions, and the focus became the people involved in the context. Subsequently, participation with some observation was planned where the researcher became an active participant in the activities of the informants.^8^ All of this information was registered in a field diary resulting in 78 field notes. The observation and the interviews were done simultaneously due to the data collection method.

The interviews were audio-recorded in Portuguese, transcribed and the data collection instrument included questions related to the characterization of the participants and open-ended questions related to representative situations of comfort care, namely comfort-promoting strategies mobilized by health professionals and particular moments of comfort and discomfort. Prior to the interview, the location, time, and date were agreed on. When interviewing professionals, we took into account their occupations, so that we would not interfere in the care of people hospitalized in the unit. When interviewing patients, the interviews were carried out either in the patient’s own unit or in a private environment. During the interview, the main researcher tried to make it flow spontaneously, following the flow of the interviewee’s ideas. We previously carried out the legitimization, clarifying doubts, and requesting authorization to turn on the recorder. All interviews were coded, based on the professional category and the number we attributed to it. The time of each interview was variable; between 30 and 45 min for health professionals and between 40 and 60 min for the person at the end of life and the family, this variation in time is due to the fact that patients and families are not conditioned in terms of time.

We organize a categorization system with all the data, this being a work of discovery, with the objective of selecting and organizing the data for further analysis.

### Data analysis

In ethnography, data analysis begins when data collection begins and constitutes a cyclical, reflective, systematic, and integrated process, often implying a constant reformulation of questions, thus accompanying the entire investigation process.^8,9^

This whole process implies and involves analysis, thought, and reflection, carried out around a systematic examination to find the parts and the relationships established with the whole.^8,9^ It also allows reiterating sustained cultural patterns in the description of cultural behaviors, artifacts and cultural knowledge.

As we collected the data, we proceeded to organize and systematize it to proceed with its codification, systematic comparison and analysis to find similarities and differences, with a view to defining phrases that could be constituted of meaning. The process involves thought and reflection, carried out around a systematic examination to find the parts and the relationships established with the whole.^9,11^ It also makes it possible to reiterate cultural patterns sustained in the description of cultural behaviors, artifacts and cultural knowledge. An attempt was made to carry out a progressive analysis that allowed the discovery of beliefs, values and practices that underlie the culture of care. To meet these standards and subsequently the domains, categories, subcategories, and sub-subcategories, we analyzed various types of data, from the field diary and unit documents (medical and nursing records, specific notes about comfort in the clinical process of patients), as well as data from interviews.

Continuing the analysis, the guiding principle was to meet the recommendations of Spradley: We reviewed and identified domains based on the information we had; observed similarities and relationships between concepts, which we incorporated into categories and subcategories, which we reorganized; identified new categories, subcategories, and sub-subcategories that led us to the reflection recommended by Spradley on ethnographic detail; created broader domains that included previously analyzed structural issues and data from all sources; resorted to the *NVivo 12* Plus program and started to organize, articulating categories and subcategories. This application made it possible to store and organize the different sources, categorize and classify qualitative data. This choice also allowed us to easily cross data, map ideas, and explore relationships between the actors.

### Methodological rigor

A research question must be clear and focused and supported by a strong conceptual framework, both of which contribute to the selection of appropriate research methods that enhance trustworthiness and minimize researcher bias inherent in qualitative methodologies.^12^ Qualitative data collection and analyses are often modified through an iterative approach to answering the research question. Researcher reflexivity, essentially a researcher’s insight into their own biases and rationale for decision-making as the study progresses, is critical to rigor.^12^ The analysis of the data itself was carried taking into account the above, by having checks and balance built into the methodology.

The work supervisor and another researcher with knowledge of the ethnographic method, added a nonbias approach. The *corpus* (composed of the field diary and all the interviews carried out) was read and revisited repeatedly throughout the entire analysis by the included researchers. We wanted to follow a scientific and rigorous method that allowed the organization, division, synthesis, and search for some patterns in the data to be analyzed independently, guaranteeing a varied and comprehensive analysis, not conditioned to the understanding of a single researcher. Comparison sessions of data analysis were carried out by the three researchers involved, ensuring the necessary triangulation in an analysis of this nature.

With regard to the topic, the use of various participants and different perspectives (person at the end of life, family and professionals) was also an important tool to ensure *credibility.* During the fieldwork, we sought to validate with the informants the contributions we were collecting, trying to understand whether our results were, in fact, meeting their experiences.

To overcome the methodological limitations, in addition to what was already mentioned after the analysis, we discussed the results with the unit’s coordinating nurse and with the team of professionals, since they could observe what Leininger alluded to as the *meaning in context*.^13^ These actors either knew the study, helping with the analysis of the relationship between domains and categories, or knew the context, which allowed the discussion of results, to understand if they were logical and legitimate.

On the other hand, we observed *saturation* and only finished collecting data when we found repeated patterns (with regard to patients and family members since all professionals were interviewed). *Transferability* was also observed, carrying out rigorous reports to allow the transfer of knowledge based on the results, to other care situations that have similar cultural conditions. It should also be noted the conversations/sharing of information with other researchers about the methodological route, the results, and the bibliography in an attempt to consolidate *fidelity*. This sharing allowed the crossing of routes and results with other members of the scientific community besides the researchers.

## Results/discussion

There were 57 participants in the study, including patients, families, and professionals. The sample consisted of 18 patients at the end of life; 18 family members (for each patient, the matched significant family member were interviewed); and 21 health professionals working with participating patients in the palliative care unit. The minimum age of the people interviewed was 26 years old and the maximum age was 94 years old. Regarding the patients, their average age was 63.8 years. The lowest aged person was 34 years old and the highest aged person was 94 years old. We also found that, with regard to the ‘gender’ variable, the distribution corresponded to exactly half of each gender, nine men and nine women. Table 1 presents the characterization of the participants.

**Table 1.** Characterization of the participants.

Group	Age	Gender	Religion	Relation to the PEL	Professional activity
Health Professional	64	Male	N/A	N/A	Spiritual assistant
Health Professional	40	Male	N/A	N/A	Operational assistant
Health Professional	29	Male	N/A	N/A	Operational assistant
Health Professional	48	Male	N/A	N/A	Operational assistant
Health Professional	53	Female	N/A	N/A	Operational assistant
Health Professional	49	Female	N/A	N/A	Operational assistant
Health Professional	43	Male	N/A	N/A	Physician
Health Professional	57	Female	N/A	N/A	Clinical director, physician
Health Professional	57	Female	N/A	N/A	Social worker
Health Professional	36	Female	N/A	N/A	Nurse
Health Professional	28	Female	N/A	N/A	Nurse
Health Professional	31	Female	N/A	N/A	Nurse
Health Professional	26	Female	N/A	N/A	Nurse
Health Professional	56	Female	N/A	N/A	Nurse
Health Professional	38	Female	N/A	N/A	Nurse
Health Professional	38	Male	N/A	N/A	Nurse
Health Professional	37	Female	N/A	N/A	Nurse
Health Professional	26	Female	N/A	N/A	Nurse
Health Professional	57	Female	N/A	N/A	Psychologist
Health Professional	38	Female	N/A	N/A	Nutritionist
Health Professional	57	Female	N/A	N/A	Physiotherapist
Family Member	73	Female	Practicing Catholic	Wife	Retired
Family Member	63	Male	Religious, nonpracticing	Husband	Carpenter
Family Member	66	Female	Religious, nonpracticing	Wife	Retired
Family Member	60	Female	Follow a religious pastor	Wife	Unemployed
Family Member	63	Female	Religious, nonpracticing	Mother	Sick leave
Family Member	61	Female	Practicing Catholic	Sister	Maid
Family Member	69	Female	Religious, nonpracticing	Wife	Unemployed
Family Member	56	Female	Religious, nonpracticing	Daughter	Supermarket employee
Family Member	49	Male	None	Husband	Attorney
Family Member	66	Female	Practicing Catholic	Wife	Retired
Family Member	44	Male	None	Husband	Bank officer
Family Member	80	Female	Practicing Catholic		Unemployed
Family Member	33	Male	Religious, nonpracticing	Son	Bank officer
Family Member	31	Female	Religious, nonpracticing	Granddaughter	Unemployed
Family Member	61	Male	Religious, nonpracticing	Ex-Husband	Accountant
Family Member	71	Female	Practicing Catholic	Mother	Retired
Family Member	70	Female	Religious, nonpracticing	Wife	Retired
Family Member	56	Female	Religious, nonpracticing	Sister	Supermarket employee
PEL	70	Female	Religious, nonpracticing	N/A	N/A
PEL	60	Female	Religious	N/A	N/A
PEL	69	Male	None	N/A	N/A
PEL	78	Male	Follow a religious pastor	N/A	N/A
PEL	34	Female	None	N/A	N/A
PEL	70	Female	Practicing Catholic	N/A	N/A
PEL	71	Male	None	N/A	N/A
PEL	79	Male	None	N/A	N/A
PEL	50	Female	Religious, nonpracticing	N/A	N/A
PEL	67	Male	Religious, nonpracticing	N/A	N/A
PEL	43	Female	Religious	N/A	N/A
PEL	64	Male	Religious, nonpracticing	N/A	N/A
PEL	60	Female	Religious, nonpracticing	N/A	N/A
PEL	94	Male	Religious, nonpracticing	N/A	N/A
PEL	55	Female	None	N/A	N/A
PEL	39	Female	Religious, nonpracticing	N/A	N/A
PEL	79	Male	None	N/A	N/A
PEL	67	Male	None	N/A	N/A

N/A, non-applicable; PEL, person at the end of life.

Despite the fact that most people are over 60 years of age, on several occasions, we have been able to witness hospitalizations of people much younger, which constitutes a challenge for everyone, whether because the person have not lived a long life or because of the suffering that accompanies the family members of younger relative. We found that the vast majority of people at the end of life hospitalized in the unit were based on a cancer diagnosis. In fact, all patients interviewed (n = 18) had a diagnosis of cancer, of the 18 patients, only seven did not have metastasized disease, the rest had metastasized disease in one or more location.

Regarding family members, it was possible to understand the dimension and importance that they have in the process of comforting the person at the end of life. There were 18 family members who informed this study. Table 1 shows that the majority of family members interviewed were female and that women were older (mean age of women 63 years) than men (mean age 50 years).

With regard to the degree of relation, the group of informing family members was quite heterogeneous. It is possible to verify that the most common degree of relation to the person at the end of life was the wife, mother, and sister. Other degrees included sons, daughters, granddaughters, ex-husband, or aunt.

Regarding the category healthcare professionals, it was possible to verify that the average age of the group of participants was 43 years old, the minimum age 26 years old, and the maximum age 64 years old.

Most healthcare professionals in this multidisciplinary team were women. The women on this team of professionals were on average slightly younger than the men: average age of women 43 years and average age of men 43.7 years. Throughout the fieldwork, we contacted several professionals who allowed us to observe their interventions, some stood out for their closeness and deep knowledge of the patient/family, their length of service in the unit and the hours practiced (namely the morning shift). There were nurses who preferably practiced the morning shift constituting our privileged participants by the interaction they established with patients and other professionals, the same happened with the operational assistants. In the case of the medical team, it was possible to maintain very close contact since the two doctors working in the unit worked from Monday to Friday from 8 am to 4 pm. It was also possible to verify that the 21 health professionals informing our study were distributed as follows: nine nurses, two physicians (one service director), five operational assistants, one social worker, one spiritual assistant, one nutritionist, a physiotherapist, and a psychologist.

The professionals most present in the daily life of the unit were undoubtedly the nurses, doctors, and operational assistants.

**Diagram 1.**
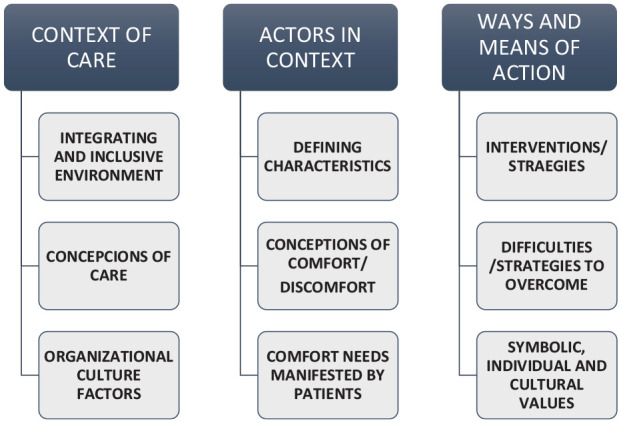
Themes and domains that shape the nature, structure, and dynamics of comfort in palliative care.

The characterization of a phenomenon implies identifying the base structure or set of conditions in which the meaning of actions and events occurs, these being fundamental determinants to understand its nature.^5,8^ The context, despite not determining the experience or fixing the action ‘identifies the sets of conditions in which the problems and/or situations respond to them through some forms of action/interaction and emotions (process) and in doing so generate consequences that, in turn, can recur and impact conditions’.^14^ The consequences ultimately determine, in this way, the outcome of actions and interactions that occur under certain conditions. Diagram 1 represents the themes and domains that inform the nature, structure, and dynamics of comforting culture and determine the general results of the main study.

From the articulation and analysis of the data, three main themes emerged that shape the comfort process in the UCP: (1) Context of Care, (2) Actors in Context, and (3) Ways and Means of action. Each of these themes contains domains that are subdivided into categories and these into subcategories and sub-subcategories that help structure and understand the results obtained.

**Diagram 2.**
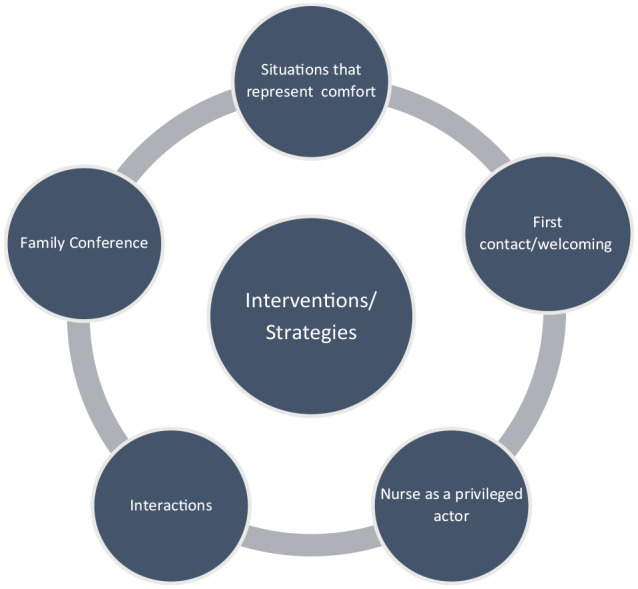
Interventions/strategies used by actors in the palliative care unit: domain and categories.

This article focuses on the results related to the ways and means of action, more specifically to the interventions/strategies. The results showed that these interventions and strategies included the following categories (Diagram 2).

We intent to show the partial results of this larger study that reveals the nurse as a privileged actor.

### Nurse as privileged actor

In this study, the nurse appears as the privileged actor of comfort. Although nursing care takes place within the multidisciplinary team and there is no doubt about the importance of complementarity between the different care actors, the recognition of the interventions performed by nurses allowed the nurse to be seen as a privileged actor of comfort, emerging in this study, as someone with a wide disposition to provide comfort. This view is shared by practically all the actors involved in this study, as we found in the content analysis, but above all by the hospitalized people ‘[. . .] they are the ones who spent most of the time with me. It starts in the morning with medication, then they help with hygiene. They provided support for my husband. They are excellent’ (PI10); ‘The massages, for example, that Nurse P. gives me with Tibetan bowls. The conversations. So many things they do that are difficult to express, but that make me very comfortable, you know?’ (PI1);

The conversations, the silences too. There was a night when I felt very anxious and afraid to fall asleep, despite having already taken the morphine, I still had that feeling, it was terrible. The nurse spent the whole night here with me in the room. We talked and even when I fell asleep she stayed there. Incredible professional and incredible human being. (PI6)

The particular attention given by nurses is widely recognized: ‘The care, the attention that nurse show. Respect for what I want and need’ (PI7); ‘There was a night when I talked about death with one of the nurses. I told her I wasn’t afraid, we talked about it without prejudice. It went really well’ (PI16). The family also highlights the role of nurses in the comfort action ‘I think that everything regarding comfort is more connected to the nurses. The conversations, the attention to detail’ (FAMI6). The family sees the nurse as a symbol of care

[. . .] the part of comfort care that nurses provide is extremely important. They are spectacular [. . .]. Symbolically, I think the issue of being a caregiver is underlying, nurses are caregivers by nature, they care about everything. Not just with the physical part, but with everything else. They listen and comfort. (FAMI4)

‘I think that in cultural terms, the issue of care is widely associate with nurses. The nurses take care of everything, as if they were octopuses, they have several tentacles and they reach a little bit everywhere’ (FAMI10).

In the context of this study, there is a multiplicity of interactions that translate into a great challenge for nurses. The interactions that took place, led by different professionals with the person at the end of life and their families, lead the nurse to a process of training/transformation that is a big challenge. It challenges the nurse to re-equate, conceptually and existentially, his or her interpretative framework. It is these constant questions that nurses ask themselves that make them look at the person in an empathetic and human way. The other members of the multidisciplinary team also recognize specific characteristics in nurses that make them privileged players in this context: ‘Nurses are the main bridge between all professionals in the unit, patients and families. They are a huge asset’ (FI2); ‘The act of comforting is the essence of nursing care. It’s their ultimate goal. During more than 30 years working in a hospital environment, I have been able to see the evolution of nursing care’ (PSI); ‘Nurses at this unit improve comfort, with a positive influence on the course of the disease’ (NTI); ‘Nurses are very empathetic and there are spectacular professionals here at this unit. Caring is closely linked to nursing’ (PCAI4).

The nurse is understood as a health professional that aims to see and understand the human being as a whole,^15^ capable of responding to the different needs of the person. Comfort can be interpreted as the experience of being supported in a multifactorial way, such as referred to in Kolcaba’s^16^ Theory of Comfort. Kolcaba^16^ defined comfort as the state in which the needs for *relief, tranquility*, and *transcendence* are satisfied The definition of comfort has grown from its most basic concept to incorporating the four contexts in which it can be experienced. The *physical* context concerns body sensations; the *sociocultural* refers to interpersonal, family, and social relationships; the *psycho-spiritual* has to do with self-esteem, sexuality, and the meaning of life/spirituality; and the *environmental* involves aspects such as light, noise, color, temperature, and elements of the environment.^16^

The relationship of the findings with the existing literature is significant, since comfort gains dimension in nursing, in an intentionally directed response and built in the interaction between the nurse and the person.

In several situations in this context, we could see that the nurse corroborates this sense of excellence in care and moves toward comforting the person at the end of life, based on the premise that human life must be preserved, always favoring the possible quality of life, the well-being and comfort of the person and family.

**Diagram 3.**
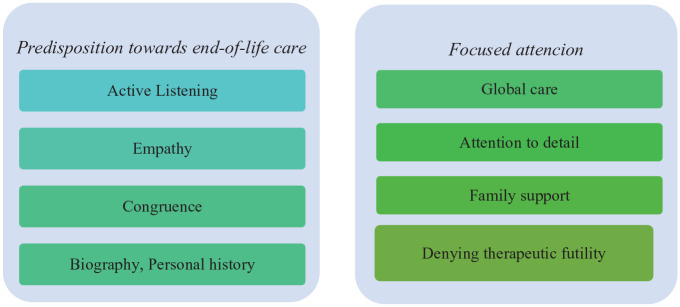
Nurse as a privileged actor in the promotion of comfort: subcategories and sub-subcategories.

In the context of this category, *Nurse as a privileged actor*, two subcategories emerged from the content analysis *Predisposition toward end-of-life care* and *Focused attention*. Despite the diversity of data sources, all converged in the sense of unanimously considering these subcategories in nurses, which are further subdivided into sub-subcategories, as outlined in Diagram 3.

The motivations and their explanations reveal a certain predisposition toward end-of-life care. This subcategory is divided into four sub-subcategories**: active listening**; **empathy**; **congruence**; and **biography/personal history**. It was evident both in field observations and in interviews that the vast majority of nurses working in this unit revealed a natural aptitude for end-of-life care and palliative patients in general. In the care process, nurses find themselves in a unique and privileged situation regarding palliative care, since they watch the patient 24 hours a day, relating more closely with the family members, being able to carry out an assessment during the provision of care. The observation also showed that the nurses develop deep communication, planning, and implementing therapeutic interventions with patients and their families.

The provision of nursing care in palliative care involves valuing all the characteristics and past experiences of the person.

It requires an attitude and approach that is not just focused on diagnosis and immediate clinical problems.

Achieving this condition requires a level of contact and humanization toward the person at the end of life that goes beyond the tasks and procedures that often dominate daily work. It is simultaneously science and art and emphasizes the feeling of normality and quality of life. It is shaped by the individual search for the meaning of life and control of vital routines with the aim of maintaining personal dignity, as the patient perceives it, in the remaining lifetime. It is an equal parts combination of knowledge, skills, and compassion. It presents itself as sensitive, hopeful, dynamic, and meaningful. However, above all, it is the philosophy of care that should guide the nurse’s behavior when caring for a person at the end of life, whatever the stage.^17^

The uniqueness among nurses in palliative care should be based on three key points: active listening, which allows accepting the word of the other, allowing the nurse to identify the needs expressed both verbally and nonverbally; the empathy that implies being able to understand the other, to put oneself in their place, while knowing how to keep the distance that allows the therapeutic relationship and congruence, which implies that the nurse must be authentic.^18^ During the observation in the field, it was possible in several situations to witness the **active listening** capacity of the nurses in this unit:

Nurses are excellent in communication, in my opinion, at least in this unit. They are excellent listeners. Respect the silences. They really listen to us so that they can understand what we need. I had never met such extraordinary nurses. (PI2)

Also in terms of field diary notes, observations were found that meet the previously mentioned.

In this context, as previously mentioned, **empathy** emerges as a sub-subcategory and is described as a characteristic of nurses by several care actors:

empathy, without a doubt, empathy! Nurses are, in my opinion, the most empathetic health professionals, at least those in this particular unit, I won’t generalize because not everyone is like that. But at this unit they are very patient, good listeners, true caregivers! (FAMI11)

The empathic component of nursing care has always been substantially valued, sustaining that to know the needs of the person, the nurse needs to try to put himself in his role, thus acting in an empathic way, as mentioned in the interview of this family member: ‘They help managing the almost unthinkable. In my case, they help to manage the fact that my daughter will die before me . . . it’s an unbearable pain, but the nurses are very empathetic, they help a lot’ (FAMI7).

The other health professionals and members of the multidisciplinary team also recognize this uniqueness in nurses with regard to empathy: ‘Well, nurses are special when it comes to that matter. The empathy that they show is wonderful. I think the underlying values are essentially empathy, kindness, willingness to help and knowing how to listen’ (PCAI5).

It was also possible during field observations and interviews to understand the **congruence** associated with the nurses’ behavior:

[. . .] The nurse enters the room and realizing that something was bothering the patient, she asked what was going on. It was the sister who answered: ‘she just wants to be in the dark, I already told her that it can’t be’. The nurse questioned the patient. If she really preferred to lower the blinds and she said yes. The nurse lowered the blinds and asked sister to come to the living room so they could talk. In the living room, firstly, she explained that the important thing is the patient’s feelings and to respect them. [. . .]. Then it was explained that the family should have an important role in promoting comfort and that this role must be integrated with the healthcare team. The sister replied that it was very disturbing for her that her sister wanted to be in the dark and began to cry. ‘She will die faster if she gives up’. The nurse considered that it would be important for the sister to gather with the other team members in a family conference without the patient, so that the whole process could be worked with the sister and to meet the needs of the sister. (FJ)

In this example, the nurse is congruent, establishing a balance between what is actually happening and the purpose she intends for the patient and her family (in this case, she intends to comfort both). The nurse in question initially ensures the comfort of the patient, which should be their priority, not neglecting the support and comfort of the sister.

There seem to be several indicators of the comforting role nurses play in different dimensions – physical, emotional, spiritual and socio-cultural, more centered on relational and communication aspects. Providing comfort makes sense when it constitutes an affective support for the person at the end of life and their family, and the human relationship proves to be fundamental in building the comfort action of nurses.

From the nurse’s point of view, the person at the end of life and their family members are the unit of care:

Comfort is the objective of palliative care [. . .] It is the essence of nursing care, reporting to mere images where the presence of nurses translates into the presence of touch, the constant daily presence, so as nurses we have to be coherent and congruent with what our functions and our essence are, especially in this area. (NI9)

This view reflects a person-centered approach, which highlights the nurse’s sensitivity to the uniqueness of each person and each family: ‘comfort is something personal. Me being comfortable does not mean that someone else is comfortable in similar conditions. It is necessary to adapt to the person, what he needs and wants’ (NI2).

When it comes to the sub-subcategory **Biography/Personal history**, the use of biographical data and some aspects of the nurses’ life history allowed access to some revelations that are related to ideas, beliefs, values, and experiences. The narrative of each nurse’s biographical data allowed for a deeper analysis of their lives and personal perspectives. What happens in nurses’ lives influences and determines their perspective. Founding experiences are established throughout life and constitute important bases that enrich the motivation for comforting care.^19^

By analyzing the nurses’ biographical data collected at the beginning of the interviews, it was possible to understand that many of them found motivation to work in this unit supported by elements, experiences and personal experiences:

I accompanied my mother’s mother who had an ovarian neoplasm, then chemotherapy, she was operated on and ended up dying in the hospital . . . and this was in the year that I entered nursing college, probably, it had something to do with it. Then my mother’s father also got sick and passed away, I had a series of end-of-life contexts on a personal level. (NI1)

My grandmothers died at home, close to us, with the family very present, establishing times and schedules to take care of them. Also a great friend who ended up passing away with breast cancer that was discovered during her pregnancy. (NI8)

Lived experience represents the way human beings experience and exist in the world as unique, and the meanings attributed to experienced situations translate the way they face life itself, experiences transform people into what they are and what they will become.^14^ Anchored in their life experiences and the relationship they established with the death process, the nurses in this context report that these same experiences had a direct influence on the choice to work in this unit: ‘On a personal level my grandparents passed away at home, then interest grew and it became my area of   choice. Coming to work at this unit was absolutely intentional’ (NI8).

The vast majority of nurses interviewed said they had great respect for this area. Of all the nurses interviewed, only one reported not having any training in the area and only two stated that the choice of working at this unit was not intentional, but rather random.

Alongside the predisposition toward end-of-life care, the motivations and the various reasons that come together also make sense in the second emerging subcategory: *focused attention* of the nurse toward the patient. After analysis, we divided this subcategory into four sub-subcategories: **global care**; **attention to detail**; **family support**, and **denying therapeutic futility.**

It seems clear that nurses benefit from particular opportunities to give their practices a comforting sense, in a multifocal way^20^ and their main attention is aligned with the provision of **global care** to the person at the end of life: ‘There is the physical, the psychological, the emotional, with the 3 in balance the person is comfortable, if there is one that is not well, the person will probably not be able to have full comfort’ (NI1); ‘A patient who is serene, calm, at ease with himself and the team, is in control, without pain’ (NI6). Focusing nurses’ attention on global care brings with it the need to recognize the patient in their uniqueness and in a cultural context that is their own:

A drop in the ocean will always make a difference. The ocean is made of all the drops, isn’t it? I believe that several aspects are considered, such as religion, personal tastes, food tastes, everything, so that the patient feels less vulnerable to the disease. (NI1)

These reports are in line with the evidence described in the literature which states that for nurses, the promotion of the humanization process consists of concern and affective involvement with the other, it depends on variables such as affection, hope, sensitivity, and comfort.^15^ Throughout the interviews, the nurses described several situations in which **the attention given to details** made a lot of difference to the patient: ‘At New Year’s Eve we had 2 patients who really enjoy this time of the year. So we planned a whole party and it was very pleasant. We barely remembered that we were at the hospital’ (NI8);

We performed relaxation measures, I stayed with the patient until she finished eating the yogurt. Clearly, she needed to talk: she spoke all evening about her life, her marriage, the death of her husband, all the family dynamics and the relationships between them, talking about how lonely she felt, I stayed with her until she fell asleep. Since then, she often says that I saved her life that night. (NI9)

The overall meaning of care from the nurse’s perspective extends to the family and there is a very significant concern with this group of people (family). Thus, another sub-subcategory that stands out from the analysis is the family support. The person represents the individual, the family and the cultural group and cannot be dissociated from their ‘cultural baggage’, since the nursing intervention must be in accordance with cultural values. It is the competence of nurses to build a care process centered on the person.^20^

At the same time, emotional support for the family finds its meaning in the concerns of nurses: ‘I think it is fundamental for the family to be supported, helped, included’ (NI3).

It appears in the nurses’ reports that emotional support to the family aims to transmit security, to be present, which in fact also meets the needs expressed by the patient, as previously mentioned. The aim is to create a positive, warm, concerned environment, based on a relationship of trust, considered a positive value and determinant in the quality of nursing care.

It was also possible, during the interviews, to perceive that some nurses felt a strong need to be transferred to the palliative care unit because they were working in units where therapeutic futility and excessive measures prevailed on the day-to-day, which led to the sub-subcategory **denying therapeutic futility**. Even with medical advances that extend survival, there is no need to consider prolonging the process of dying at any cost, despite the freedom and autonomy of the person to actively participate in decisions related to their treatment. Therapeutic futility occurs when there are unnecessary extensions, which will not bring benefits to the terminally ill person. In these situations, due to a lack of familiarity with all aspects involving death, the health professional may experience a feeling of impotence and frustration. However, nurses have proven themselves to be very helpful to these patients and their family members.^21^

The tendency toward the humanization of the relationship between the nurse and the patient is getting more and more consistent since it is absolutely essential to assure comfort for the patient. The nurse has communication skills as the foundation of collaborative care centered on the person and family, and not on the doctor or the disease, as in the past.^17^ Some of the nurse’s interviews mentioned that these issues related to therapeutic relentlessness caused them some anxiety in units they have worked previously:

‘During the time I was in Internal Medicine, I was in the presence of palliative patients, not always treated as such. However, I started to feel the need to look for more things to help these patients at the end of life because I thought that we were doing things that didn’t make sense at this point’ (NI1); ‘In the Medicine unit I also had contact with people at the end of life, although it is a different “end of life”. Here in the Unit it is a “programmed end of life”, we know that it will happen in the near future while in the Medicine unit it was either not expected (acute reasons) or they died in another way, not palliative’ (NI2).

## Conclusion

In this article, we chose to highlight the determinants that seem to be related to the fact that nurses are considered privileged actors of comfort: the *predisposition for end-of-life care* and the *focused attention* of nurses. Thinking about the meaning they give to the activities they perform; we find what motivates and concerns nurses in the practice of end-of-life care. Based on an enriched view of care, the nurse also recognizes and values him/herself as a primordial actor of comfort.

Nurses are also healthcare professionals who, in addition to specific training, have greater holistic knowledge of people/families and mobilize strategies that demonstrate well-being to optimize care, guaranteeing care through the constant presence and functioning as a fundamental actor of interconnection between all the elements of the team. ‘Nurses are the main bridge between all professionals in the unit, patients and families. They are a huge asset’ (FI2). Behavior is guided by desires, motivations and needs that guide performance. The nurse is consistent in the knowledge, in the capacities and competencies mobilized in comfort care and in the reflection on actions, which makes it possible for him or her to (re)adopt appropriate strategies conducting to the promotion of comfort. An incorporated knowledge of values and experiences is evident, guiding determinants in the professional trajectory, in a permanent articulation between the heritage of individual and collective meanings.^4,5^

The nurse’s role thus assumes a very significant and special dimension in comforting care. We found that nurses benefit from a particular way of acting that they seek to give to their practice and which is embodied in a relationship of empathy. The nurse seeks to be with the patient, meet him and understand him. It takes place in a sense of real and effective help, with the nurse acting as a vector of comfort for people at the end of life and their families.

We consider that the research carried out provided several contributions to the provision of care to the person at the end of life and it is hoped that it will be a stimulus to scientific research in the area of palliative care, which could be structuring and inspiring for questioning and reformulating practices related to the management of nursing care and nursing resources, in the current situation. We can also consider, with regard to implications for practice, that looking at palliative and end-of-life care with a central focus, for nurses, is a process that begins on the bases of knowledge. It is necessary to develop the concept of comfort in initial training as well as in postgraduate training in nursing, to understand what underlies it so that one can think and act within an individualized intervention framework, where the intervention design takes place, but also the implementation and the evaluation/analysis of the obtained results.

### Limitations of the study

Most of the data collection took place during the pandemic period, which created several obstacles, namely, the creation of a *focus group*, which was not possible, due to the need to reduce the risks of contamination at that time.

Regarding the limitations of the study findings, since we intended to study the nurse as a privileged player in this context we think that there might be a poor representation of nurses in the study (only 9 nurses worked at this unit).

However, knowing that each ethnographic study cannot be ‘reproduced’, these results can serve as a guide for building and consolidating knowledge about a phenomenon considered noble and associated with nurses’ interventions.

## Declarations

**Ethics approval and consent to participate:** The study was conducted according to the guidelines of the Declaration of Helsinki and approved by the CHBM Ethics Committee and Universidade Católica Portuguesa de Lisboa Ethics Committee. Approval number or ID provided by the CHBM Ethics Committee and Universidade Católica Portuguesa de Lisboa Ethics Committee: Memorando Nota Interna: 48/2019.

**Consent for publication:** Participants provided written informed consent prior to the start of data collection.
